# Actomyosin and vimentin cytoskeletal networks regulate nuclear shape, mechanics and chromatin organization

**DOI:** 10.1038/s41598-017-05467-x

**Published:** 2017-07-12

**Authors:** Michael C. Keeling, Luis R. Flores, Asad H. Dodhy, Elizabeth R. Murray, Núria Gavara

**Affiliations:** 1 School of Engineering and Materials Science, Queen Mary University of London, Mile End Road, E1 3NS London, UK; 2 Kinase Biology Laboratory, John Vane Science Centre, Barts Cancer Institute, Queen Mary University of London, Charterhouse Square, London, EC1M 6BQ UK

## Abstract

The regulation of nuclear state by the cytoskeleton is an important part of cellular function. Actomyosin stress fibres, microtubules and intermediate filaments have distinct and complementary roles in integrating the nucleus into its environment and influencing its mechanical state. However, the interconnectedness of cytoskeletal networks makes it difficult to dissect their individual effects on the nucleus. We use simple image analysis approaches to characterize nuclear state, estimating nuclear volume, Poisson’s ratio, apparent elastic modulus and chromatin condensation. By combining them with cytoskeletal quantification, we assess how cytoskeletal organization regulates nuclear state. We report for a number of cell types that nuclei display auxetic properties. Furthermore, stress fibres and intermediate filaments modulate the mechanical properties of the nucleus and also chromatin condensation. Conversely, nuclear volume and its gross morphology are regulated by intracellular outward pulling forces exerted by myosin. The modulation exerted by the cytoskeleton onto the nucleus results in changes that are of similar magnitude to those observed when the nucleus is altered intrinsically, inducing chromatin decondensation or cell differentiation. Our approach allows pinpointing the contribution of distinct cytoskeletal proteins to nuclear mechanical state in physio- and pathological conditions, furthering our understanding of a key aspect of cellular behaviour.

## Introduction

The nucleus is the largest and stiffest organelle in eukaryotic cells and it contains its genetic material. The mechanical behaviour of the nucleus and its role in cellular mechanotransduction depends on multiple factors, including its own makeup and that of its environment. Intrinsically, the expression of laminA/C (type V intermediate filaments) and the organization of chromatin are believed to determine the mechanical properties of the nucleus^1^. Externally, cytoskeletal networks, notably actin, have also been suggested to play a key role in nuclear state and mechanics^2^. Nuclear stiffness has been shown to scale with tissue stiffness^3^, as well as dynamically increase along the course of stem cell differentiation^4^. Abnormal nuclear shapes and mechanical properties are linked to cancer and aging^5^. Furthermore, aberrant laminA/C expression leads to a host of diseases known as laminopathies, which are associated with altered chromatin organization^6^. Though the importance of nuclear morphology and mechanics for correct cell and tissue function is slowly being revealed, the mechanism of action still remains to be characterised.

The morphology and mechanical properties of the nucleus change dynamically, allowing it to perform in different mechanical environments. Migrating cells are able to reorganise their nuclear material, enabling their passage through pores as narrow as 10% of the nuclear diameter^7^. In addition, when external mechanical loads are applied onto cultured cells using AFM colloidal tips, nuclei can be compressed up to 20% of their height in a reversible manner and without apparent damage^8^. Recently, it has been proposed that, under certain conditions, nuclei exhibit auxetic characteristics, that is, their Poisson’s ratio is negative^9^. When auxetic materials are stretched in one direction, they also increase in size in the directions perpendicular to the applied force. This mechanical behaviour has been typically observed in foam structures and, in the case of cellular nuclei, has been partly attributed to chromatin decondensation^9^.

The nucleus is integrated into the mechanical milieu of the cell through specific proteins composing the LINC complex^10^. This allows forces at the cell membrane to be transmitted via the cytoskeleton to deform the nucleus^11^ and intranuclear structures^12^. The mechanical interplay between the cytoskeleton and the nucleus has been best characterised for actin, while the influence of other cytoskeletal networks is not as widely studied or understood. A body of research has shown that stress fibres couple nuclear shape to cell shape^13, 14^ and are involved in modulating nuclear morphology of differentiating cells^15^ as well as the organisation of laminA/C^16^. On the other hand, the microtubule network has been shown to support nuclear rotation and repositioning, highlighting its mechanical link with the nucleus via microtubule-associated motor proteins and members of the nesprin family^17, 18^. Finally, the intermediate filament network has been recently proposed as an additional player in the mechanical regulation of nuclear shape in keratin-rich cells^19^. It should be pointed out that the findings obtained in the studies presented so far have been based on the use of knock-out cell lines or the selective inhibition/depolymerisation of cytoskeletal proteins of interest. Nevertheless, the elements of the cytoskeleton do not exist in isolation inside the cellular milieu, instead they are intimately linked physically as well as in their co-regulation. Accordingly, disruption of one network is likely to alter the organization and mechanical state of another and vice versa^19, 20^. In a similar fashion, the popular use of micropatterning to modulate the actin cytoskeleton by limiting cell spread area^13, 21, 22^ fails to acknowledge that the organization of the other cytoskeletal networks is most likely disturbed too. Taken together, these limitations make it difficult to confidently attribute specific changes in nuclear morphology, mechanics or chromatin organization to a specific cytoskeletal network, rather than to global changes of cytoskeletal state. Untangling the mechanical contribution of each cytoskeletal network to nuclear shape and mechanics thus remains an unsolved issue if we are to understand the role of nuclear mechanotransduction in cell fate and function.

In the present study we have assessed the individual contribution of cytoskeletal networks (actin, microtubules and intermediate filaments) in the modulation of nuclear morphology and mechanical behaviour, as well as the level of chromatin condensation. To do so, we have used a simple image quantification approach to readily estimate three-dimensional nuclear shape, mechanical properties and chromatin condensation using low magnification epifluorescence imaging. We combine this method with image quantification algorithms previously developed by us to quantify cytoskeletal organization^23^. The simultaneous use of both image analysis methods allows us to correlate, at the single cell level, cytoskeletal organization with a number of relevant parameters to describe nuclear state, namely nuclear volume, Poisson’s ratio, apparent elastic modulus and chromatin condensation. Our results highlight the role of actomyosin stress fibres and vimentin intermediate filaments as key players in the mechanical regulation of nuclear state by the cytoskeleton. Furthermore, the regulation exerted by the cytoskeleton on the nucleus gives rise to morphological and mechanical changes that are of the same magnitude than those attained by altering it intrinsically, such as by using Trichostatin A (TSA) to decondense chromatin or by inducing cells to differentiate. Our results place the cytoskeleton in the centre stage of mechanical regulation of cell behaviour and fate, not only by its long-established role as mechanotransducer and load-bearing structure, but also due to its major influence in nuclear state.

## Results and Discussion

### Cells with larger spread areas display more flattened nuclei

To understand the mechanical relationship between cytoskeletal networks and the nucleus, we have chosen human mesenchymal stem cells (hMSC) as primary cellular model. When well-spread, hMSCs display marked actomyosin stress fibres that can be easily imaged and quantified using our algorithms. Similarly, microtubules and intermediate filaments are also clearly observed and readily quantified. hMSC are vimentin-rich and contain no keratin, thus simplifying the characterization of the role of intermediate filaments. Finally, the possibility to induce hMSC differentiation using well-characterized osteogenic and adipogenic media allows us also to compare the magnitude of CSK-mediated regulation of the nucleus versus plastic changes in nuclear morphology and chromatin condensation, such as those experienced by cells during differentiation.

**Figure 1:**
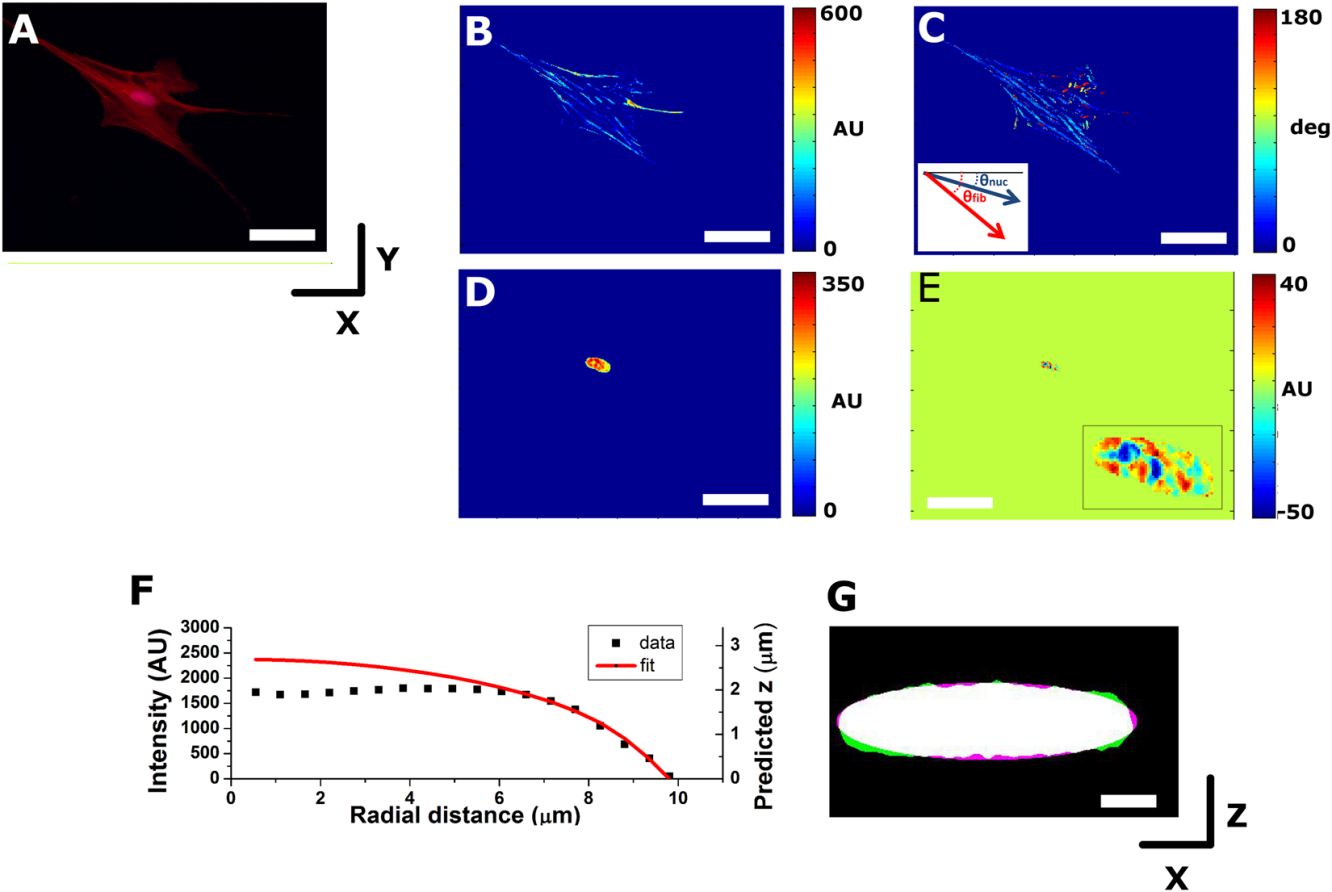
Epifluorescence–based quantification of cytoskeletal organization, nuclear shape and chromatin condensation. All panels depict the same example cell/nucleus. (**A**) Overlay of fluorescence images for TRITC (phalloidin) and DAPI channels obtained on an epifluorescence microscope using a 20× objective. Quantification of F-actin fibre fluorescence intensity (**B**) and fiber orientation (**C**) obtained from the raw images. Fluorescence intensity of the nucleus before (**D**) and after (**E**) band-pass filtering. Fluorescent speckles resulting from areas of high chromatin condensation are clearly visible in the zoomed-in image shown as an inset. (**F**) Averaged fluorescence intensity profile as a function of radial distance I(r). Black squares correspond to fluorescence intensities recorded, and the imaged nucleus is taller than the depth of focus of the objective lens. Red line corresponds to the ellipse obtained when fitting the fluorescence intensity profile of the outermost pixels. Left axis shows the fluorescence intensity values from the analysed image, while right axis shows the height profile estimated using the calibration factor. (**G**) x-z reconstruction of the nucleus as obtained from a confocal image stack. Overlaid is the estimated gross nuclear morphology as obtained from the fit shown in (**F**). Scale bar is 50 µm in panels A–E and 5 µm in panel G. In panels B–E a false colour scale has been used to improve visualization. Inset in (**C**) exemplifies the computed orientation of the nucleus (θ_nuc_) and the average orientation of the fibres (θ_fib_).

**Figure 2:**
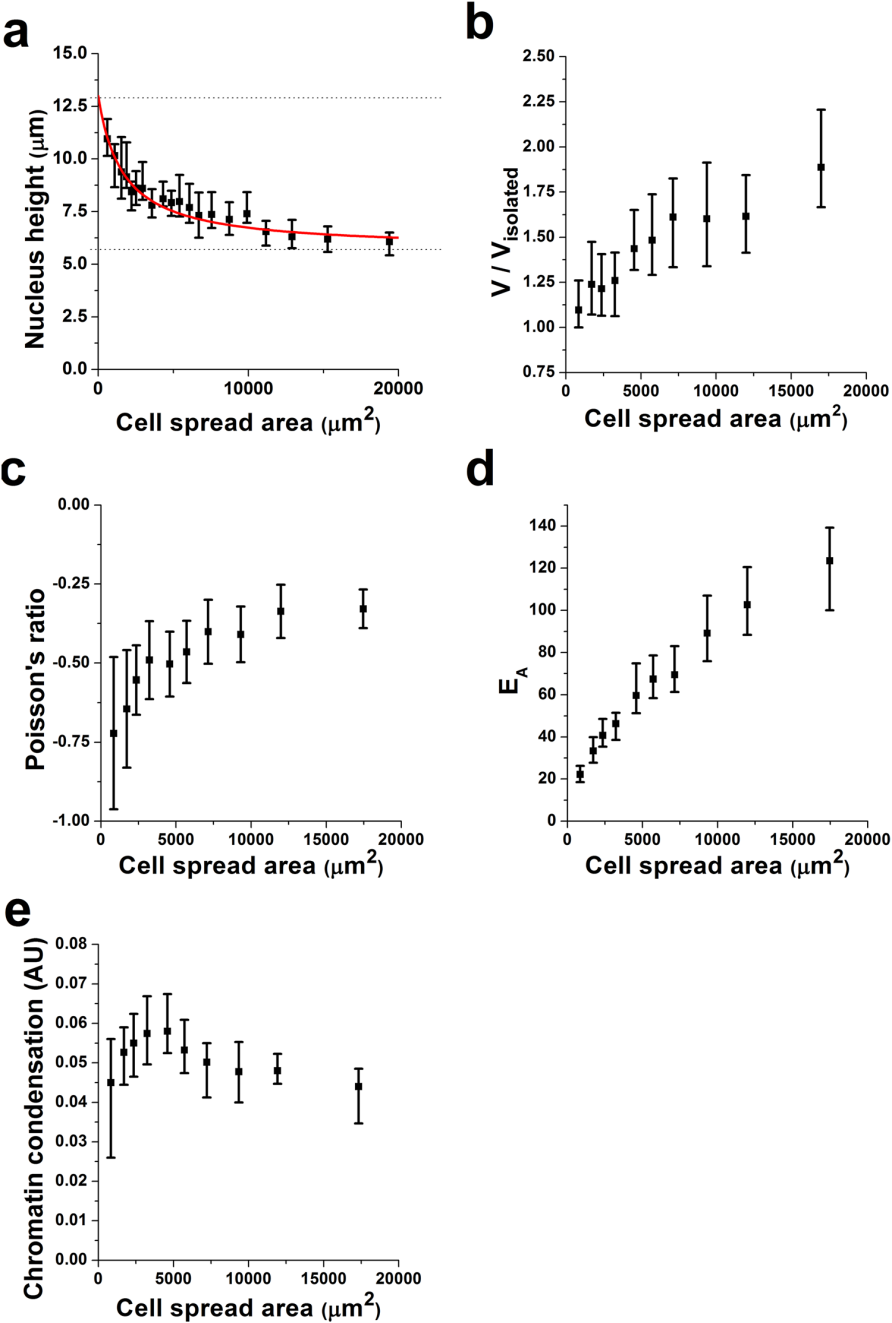
Cell spread area modulates nuclear state and mechanics. Plot shows results for nuclear height (**a**), volume (**b**), Poisson’s ratio (**c**), apparent elastic modulus (**d**) and chromatin condensation (**e**). Values for >50 cells were pooled to compute each individual data point. Data is presented as geometric mean, error bars indicate interquartile range (Q1–Q3). For the single case of Poisson’s ratio, data is presented as mean ± SD. For height data, the red line is the fit to the rational function defined as *h* = $a+\frac{b}{c+CSA}$. Dotted lines indicate the values for average height of isolated nuclei and minimum nuclear height, as estimated using the fit.

To obtain a large range of cellular and cytoskeletal morphologies we have used cells cultured at very low density in unrestricted spreading conditions, taking advantage of their inherent variability. In our experiments, cell areas ranged between 843 and 18,000 µm^2^, with a median value of 5,200 µm^2^. Firstly, we measure nuclear height using our simple 2D imaging approach to estimate nuclear 3D gross morphology (Fig. 1, see also Supplementary Method). We find that cell spread area has a marked effect on the observed nuclear height, with well-spread cells displaying much more flattened nuclei (Fig. 2A). We fit our data to a rational function such as *h* = $a+\frac{b}{c+CSA},$ were *h* corresponds to nuclear height and CSA indicates cell spread area. This is a convenient fit because its bounds allow us to predict the two extremes of nuclear shape. On the one hand, we compute the height displayed by nuclei in isolated conditions (*h*_*i*_), imposing CSA = 0. On the other hand, we compute the compressibility limit, that is, the minimum thickness that nuclei display when they can’t be compressed further, imposing *CSA* → ∞. For the average nuclear height in isolated conditions, we obtain a value of 12.9 ± 0.4 µm, in agreement with previous estimates^24^. For average minimum nuclear height, we estimate a value of 5.7 ± 0.1 µm. Together, these results indicate that during physiological processes associated with cell adhesion and spreading, nuclei may be compressed in the axial direction up to 44% of their height in isolated conditions. This estimate is smaller than values obtained by others using a colloidal AFM tip to apply compressive forces onto nuclei of adherent cells, where nuclei were compressed down to 19% of their initial height^8^. Of note, the estimate we obtain is based on nuclei flattened by intracellular forces only, thus highlighting instances when no external mechanical load is present. Conversely, when very stiff AFM cantilevers are used to attain much larger compressive forces (up to 800 nN were applied in ref. 8), the probing conditions allow exploring the limits of the nucleus as a biological material subjected to extreme deformations.

### Cell spread area also modulates nuclear volume and nuclear mechanical properties

Having estimated the nuclear dimensions for isolated nuclei, we moved on to assess how cell spread area regulates nuclear properties in physiological conditions. We find that the decrease in nuclear height as cell area increases is accompanied by an increase in volume, reaching values up to 80% larger than the nuclear volume in isolated conditions (Fig. 2B). Together, these results indicate that nuclei tend to be compressed in the vertical direction, but stretched in the in-plane direction, which is the direction typically displayed by stress fibres. While the ionic composition of the cytoplasm could cause the nucleus to swell, the fact that we and others find that nuclear volume increases with cell area^21^ primarily due to elongation in the in-plane direction rather points to the nucleus being subjected to intracellular outward pulling forces, as previously suggested by others^25, 26^. When measuring the mechanical properties of the nucleus, we find that the apparent elastic modulus increases with increasing cell area (Fig. 2D), thus confirming experimentally the prediction of a previous study^14^. Nuclear Poisson’s ratio, that is, the compressibility of the nucleus, was also strongly influenced by cell area (Fig. 2C). Interestingly, nuclei displayed negative Poisson’s ratio, thus corresponding to the type of materials classed as auxetic. Such materials don’t display conservation of volume when subjected to deformation, but rather tend to shrink when subjected to compressive forces, or swell when subjected to tensional forces^27^. Isolated nuclei and nuclei of non-adherent cells (a situation which would correspond to the leftmost region in Fig. 2) have been shown to be auxetic^9^. Thus our findings extend the range at which nuclei have been observed to be auxetic into more physiological scenarios.

### The cytoskeleton as the underlying driver of nuclear shape and mechanics

**Figure 3:**
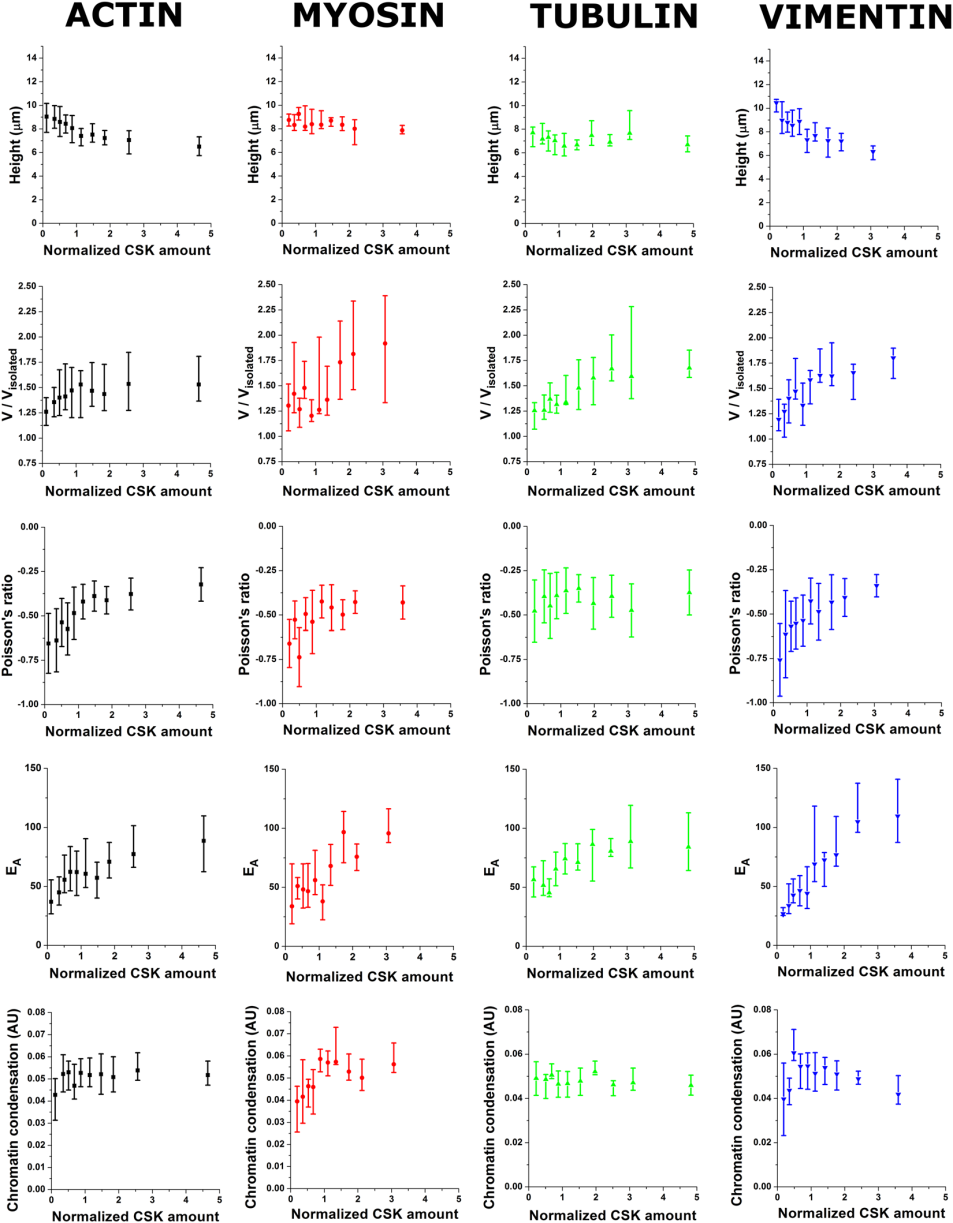
Cytoskeletal organization modulates nuclear state and mechanics. Subplots are arranged according to cytoskeletal protein assessed (*columns*) and nuclear property measured (*rows*). Values for >15 cells were pooled to compute each individual data point. Data is presented as geometric mean, error bars indicate interquartile range (Q1–Q3). For the single case of Poisson’s ratio, data is presented as mean ± SD.

We next hypothesized that the relationship we observe between nuclear properties and cell spread area is an external manifestation of the fact that cytoskeletal organization modulates nuclear state. Taking advantage of our cytoskeletal quantification capabilities, we plotted nuclear shape and mechanics as a function of cytoskeletal assembly for actin, myosin, tubulin and vimentin (Fig. 3). Of note, we still find relationships that resemble those previously found for cell spread area. Nevertheless, the trends obtained are stronger for certain proteins than others. In particular, vimentin and actin display the strongest trends in the case of Poisson’s ratio and apparent elastic modulus, while vimentin and myosin appear to strongly influence nuclear volume. Finally, microtubule assembly appears to have a weak but consistent influence in most nuclear parameters, displaying similar trends to all other cytoskeletal proteins but in a weaker way.

**Figure 4:**
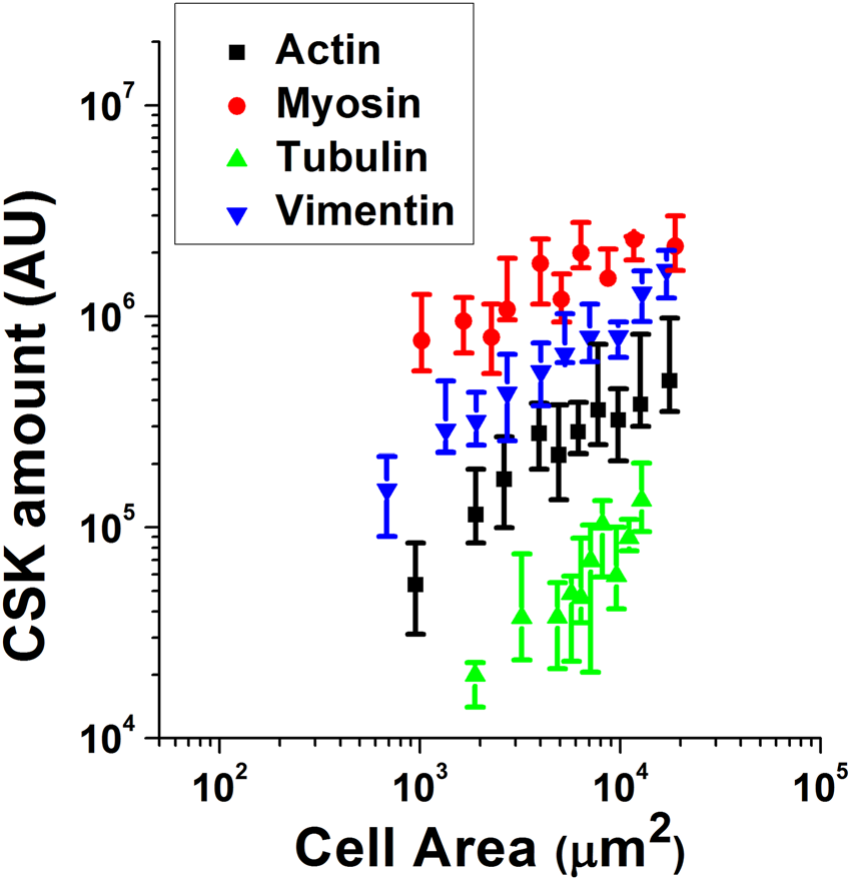
Cell spread area modulates cytoskeletal organization. Plot shows cytoskeletal organization in filamentous form for actin (*black*), myosin (*red*), tubulin (*green*) and vimentin (*blue*). Values for >15 cells were pooled to compute each individual data point. Data is presented as geometric mean, error bars indicate interquartile range (Q1–Q3).

Given that cytoskeletal polymerization of actin, myosin, tubulin and vimentin is likely interlinked with cell spread area, it should come as no surprise that all cytoskeletal networks appear to have a role in modulating nuclear mechanics. In particular, it may be hypothesized that increases in the assembly of one particular cytoskeletal network will appear alongside increases in assembly of all other networks. Accordingly, we find that cell spread area strongly correlates with cytoskeletal assembly of all measured proteins (Fig. 4)^21^. In particular, we obtain the following exponents when fitting a power law relationship [*X*] ∝ *CSA* ^*γ*^ between cell spread area and cytoskeletal filamentous assembly ([X]): γ_A_ = 0.56 ± 0.07 (*p* = 1.2e-07), γ_M_ = 0.37 ± 0.08 (*p* = 4e-06), γ_T_ = 1.05 ± 0.2 (*p* = 1.6e-05), γ_V_ = 0.76 ± 0.06 (*p* = 4e-08), where the sub-indices indicate actin, myosin, tubulin and vimentin, respectively. While the slopes are markedly different, they all fall in the weak power law regime, suggesting that the modulation of cell spread area impacts the assembly of all cytoskeletal networks, and it does so in an analogous way. Taking these results into account, we need to revisit the results found in Fig. 3, and use a different strategy to untangle the effects of each individual network on nuclear morphology and mechanics.

### Actomyosin and vimentin as the main drivers of nuclear shape and mechanics

Our results so far suggest qualitatively that some cytoskeletal proteins have a stronger influence than others in nuclear state. To pinpoint the key players in nuclear state, we have devised the following strategy, based on our capabilities to simultaneously quantify nuclear state together with cytoskeletal organization for 2 different protein networks. In brief, we use graded dosages of drugs known to induce the depolymerization of specific cytoskeletal networks (Blebbistatin at 5–50 µM to reduce Myosin activity, Withaferin A at 0.5–2 µM to depolymerize Vimentin filaments and Nocodazole at 0.5–2 µg/ml to depolymerize Microtubules). But unlike others, we analyse cells on a single-cell basis rather than pooling them into cell populations according to dosage. As expected, when a particular protein is inhibited, we observe lower polymerization levels for said protein in comparison to actin polymerization levels (Suppl. Fig. 3 top panels). In particular, when we fit polymerization levels as e.g. [*M*] = [*A*]^*β*^, we measure a drop in α values for all treatments and dosages used (Suppl. Fig. 3 bottom panels). Given these results, we assume also no compensatory mechanisms between the other cytoskeletons when inhibitory drugs are used (e.g. we assume no changes in vimentin polymerization levels when microtubules are depolymerized). When we use our single-cell approach, we notice that intrinsic cellular variability results in a number of cells for which cytoskeletal assembly of e.g. actin and myosin is largely uncorrelated (data points laying far from the average population trends in Suppl. Fig. 3a–c). This phenomenon is especially useful for us, because it allows us to untangle the mechanical effect of different cytoskeletal network in our data. Accordingly, we use all our data simultaneously (~600 cells) to correlate nuclear parameters vs CSK amount, using an iterative global fit approach (see Suppl. Methods) with the following dependencies:

1 $$E={E}_{0}{[\frac{A}{\langle \bar{A}\rangle }]}^{{\alpha }_{A}}{[\frac{M}{\langle \bar{M}\rangle }]}^{{\alpha }_{M}}{[\frac{T}{\langle \bar{T}\rangle }]}^{{\alpha }_{T}}{[\frac{V}{\langle \bar{V}\rangle }]}^{{\alpha }_{V}},$$

where *E* is here used as an example of one of the nuclear parameters measured.

Using this strategy we are able to pinpoint the specific effect of each cytoskeletal network on nuclear shape and mechanics, as presented in Table 1. We find that actin and vimentin have the largest contribution to the modulation of Poisson’s ratio, while vimentin and myosin have the strongest effect on nuclear volume. In the case of apparent elastic modulus, all networks except actin contribute to stiffen the nucleus. These contributions highlight the role of cytoskeletal structures as scaffolds of the nucleus, being mechanically connected to it and passively opposing its reorganization. This phenomenon is more understandable in the case of apparent elastic modulus, because the nucleus appears to be stiffer (it deforms less) when it is surrounded by denser networks of intermediate filaments and microtubules. Similarly, an auxetic material will appear to be less auxetic when scaffolded by a reinforced network, as the intrinsic changes in volume associated with compressive or tensile loads will be opposed by the scaffolding/caging network.

**Table 1:** Results of the global fits between cytoskeletal network assembly and nuclear parameters.

Volume	$V={V}_{0}{[\frac{M}{\langle \bar{M}\rangle }]}^{{\alpha }_{M}}{[\frac{T}{\langle \bar{T}\rangle }]}^{{\alpha }_{T}}{[\frac{V}{\langle \bar{V}\rangle }]}^{{\alpha }_{V}}$
*V*_0_	1.4323 ± 0.0026 (*p* = 0)
*α*_*M*_	0.094 ± 0.033 (*p* = 0.004)
*α*_*T*_	0.073 ± 0.019 (*p* = 4e-6)
*α*_*V*_	0.125 ± 0.016 (*p* = 3e-10)
**Poisson’s ratio**	$\nu ={\nu }_{0}{[\frac{A}{\langle \bar{A}\rangle }]}^{{\alpha }_{A}}{[\frac{V}{\langle \bar{V}\rangle }]}^{{\alpha }_{V}}$
***ν***_***0***_	−0.440 ± 0.009 (*p* = 2e-157)
*α*_*A*_	−0.152 ± 0.019 (*p* = 4e-20)
*α*_*V*_	−0.102 ± 0.027 (*p* = 2e-4)
**Apparent elastic modulus**	$E={E}_{0}{[\frac{A}{\langle \bar{M}\rangle }]}^{{\alpha }_{M}}{[\frac{T}{\langle \bar{T}\rangle }]}^{{\alpha }_{T}}{[\frac{V}{\langle \bar{V}\rangle }]}^{{\alpha }_{V}}$
*E*_0_	58.39 ± 1.29 (*p* = 0)
*α*_*M*_	0.192 ± 0.057 (*p* = 7e-4)
*α*_*T*_	0.225 ± 0.031 (*p* = 8e-11)
*α*_*V*_	0.443 ± 0.033 (*p* = 6e-31)
**Chromatin condensation**	$C={C}_{0}{[\frac{A}{\langle \bar{A}\rangle }]}^{{\alpha }_{A}}{[\frac{V}{\langle \bar{V}\rangle }]}^{{\alpha }_{V}}$
*C*_0_	0.0491 ± 0.0008 (*p* = 0)
*α*_*A*_	0.081 ± 0.011 (*p* = 0)
*α*_*V*_	−0.072 ± 0.024 (*p* = 0.001)
**Height**	$C={C}_{0}{[\frac{A}{\langle \bar{A}\rangle }]}^{{\alpha }_{A}}{[\frac{V}{\langle \bar{V}\rangle }]}^{{\alpha }_{V}}$
*C*_0_	7.80 ± 0.07 (*p* = 0)
*α*_*A*_	−0.064 ± 0.008 (*p* = 0)
*α*_*V*_	−0.078 ± 0.014 (*p* = 3e-8)

Only the fitting constants that yielded *p* values smaller than 0.05 were used for the final global fits, all other fitting constants (α_X_) were set to 0 to reduce the number of free parameters in our final models.

**Figure 5:**
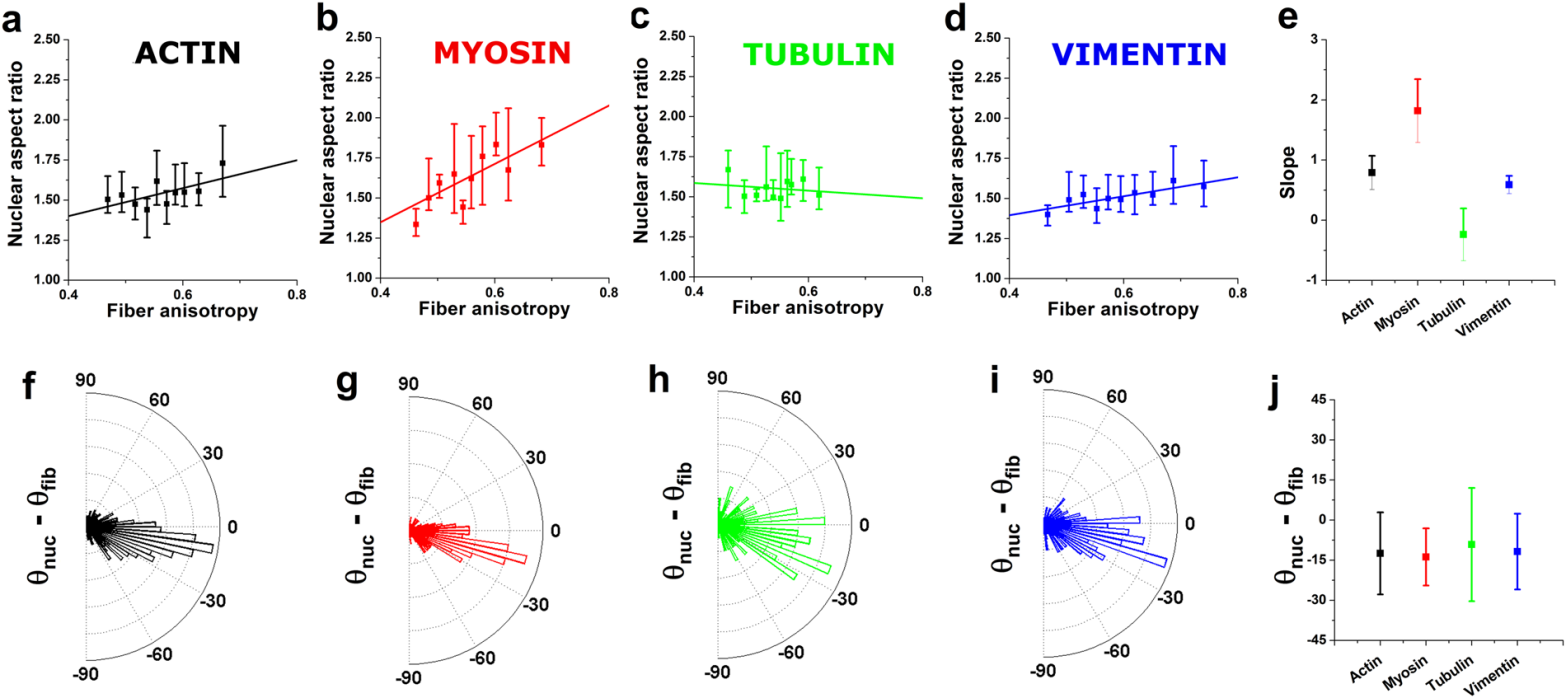
The alignment of cytoskeletal fibres affects nuclear shape and orientation. *Top row* shows the relationship between fibre anisotropy and nuclear aspect ratio in the x-y plane and *bottom row* shows the difference between the orientation of the nucleus (θ_nuc_) and that of the fibres (θ_fib_). Panels depict these relationships for actin (*black*), myosin (*red*), tubulin (*green*) and vimentin (*blue*). In the top row, values for >15 cells were pooled to compute each individual data point and data is presented as geometric mean, error bars indicate interquartile range (Q1–Q3). Panel *e* shows the slopes of the linear fits obtained for panels a–d while panel j shows the circular mean and circular standard deviation obtained from the distributions presented in **f**–**i**.

While the effects discussed above can be associated with the passive regulation of nuclear mechanics by a reinforced scaffold, the role of myosin in nuclear regulation can be associated with active mechanisms. In particular, the role of myosin on nuclear volume points to the fact that the nucleus is a cellular organelle subjected to intracellular tension^25, 26^. While others have so far focused on the effect of intracellular tension in flattening the nucleus via the application of compressive loads^13, 14^, our results highlight the role of cytoskeleton-driven outward pulling forces in the regulation of nuclear volume. In brief, our image quantification pipeline identifies fibrillar structures in immunostained images, and outputs their fluorescence intensity (Fig. 1B) as well as their direction (Fig. 1C). Therefore, we are able to measure the preferred angular orientation of fibrillar structures in a cell and compare it to the in-plane orientation of the nucleus, given by the angular orientation displayed by the major axis *a* (Fig. 1D). Figure 5 presents this results for all 4 cytoskeletal proteins tested. Of note, nuclei are preferentially aligned with the direction of myosin-rich fibrillar structures (in Fig. 5, the smallest SD on angular distributions is obtained for myosin, as shown in panels g and j). Moreover, cells with more aligned myosin-rich fibrillar structures (higher fibre anisotropy) tend to have more elongated nuclei in the x-y plane (in Fig. 5, the highest slope is found for myosin, as shown in panels b and e). Interestingly, the fact that vimentin also has a significant role in modulating nuclear volume further indicates that the intermediate filament network is required as passive scaffolding, to mechanically transmit the active pulling forces generated by myosin. Finally, our observation that increasing levels of myosin assembly make the nucleus appear to be stiffer is a fitting counterpart to results found by others, where nuclei stiffen when larger forces are applied onto them via an AFM tip^8^. Together, these results present the nucleus as an organelle that stiffens when mechanically loaded, disregarding whether the origin of the load is cell-generated intracellular tension or extracellular forces acting on the cellular environment.

### Actin and vimentin regulate chromatin condensation in an opposing manner

Having identified the role of cytoskeletal networks on the modulation of nuclear shape and mechanics, we moved on to assess whether this modulation could further affect intranuclear structures such as the organization of chromatin. First, we confirm a strong correlation between nuclear volume and chromatin condensation, with larger nuclei displaying decreased levels of condensation (*CC* ∝ *βV*, β = −0.013 ± 0.003; *p* = 0.0003). When observing the effect of cell area on chromatin condensation (Fig. 2E), the behaviour is less straightforward than that displayed by other nuclear parameters. In particular, chromatin condensation appears to peak for spread areas ~3,500 µm^2^, while cells with smaller or larger cell spread areas have lower levels of chromatin condensation. Of note, a similar behaviour is found if we measure chromatin condensation only at the nuclear periphery, corresponding to the areas where heterochromatin is preferentially located (not shown). When assessing the effect of individual networks (Fig. 3 and Table 1), we begin to identify the opposing effects of actin and vimentin on chromatin state. Interestingly, our results suggest that higher levels of actin assembly will tend to condense chromatin, while higher levels of vimentin will tend to decondense it. The final outcome for these two opposing effects is determined by the magnitudes in which actin and vimentin filament assembly increase with increasing cell area. As presented in section 2.3, for the cell type and culture conditions discussed here, vimentin assembly increases more than actin assembly as cells spread (γ_A_ = 0.56 ± 0.07 vs. γ_V_ = 0.76 ± 0.06). Accordingly, the chromatin decondensing role played by vimentin dominates for large cell spreading areas.

### Cytoskeleton-mediated versus internal regulation of nuclear state

**Figure 6:**
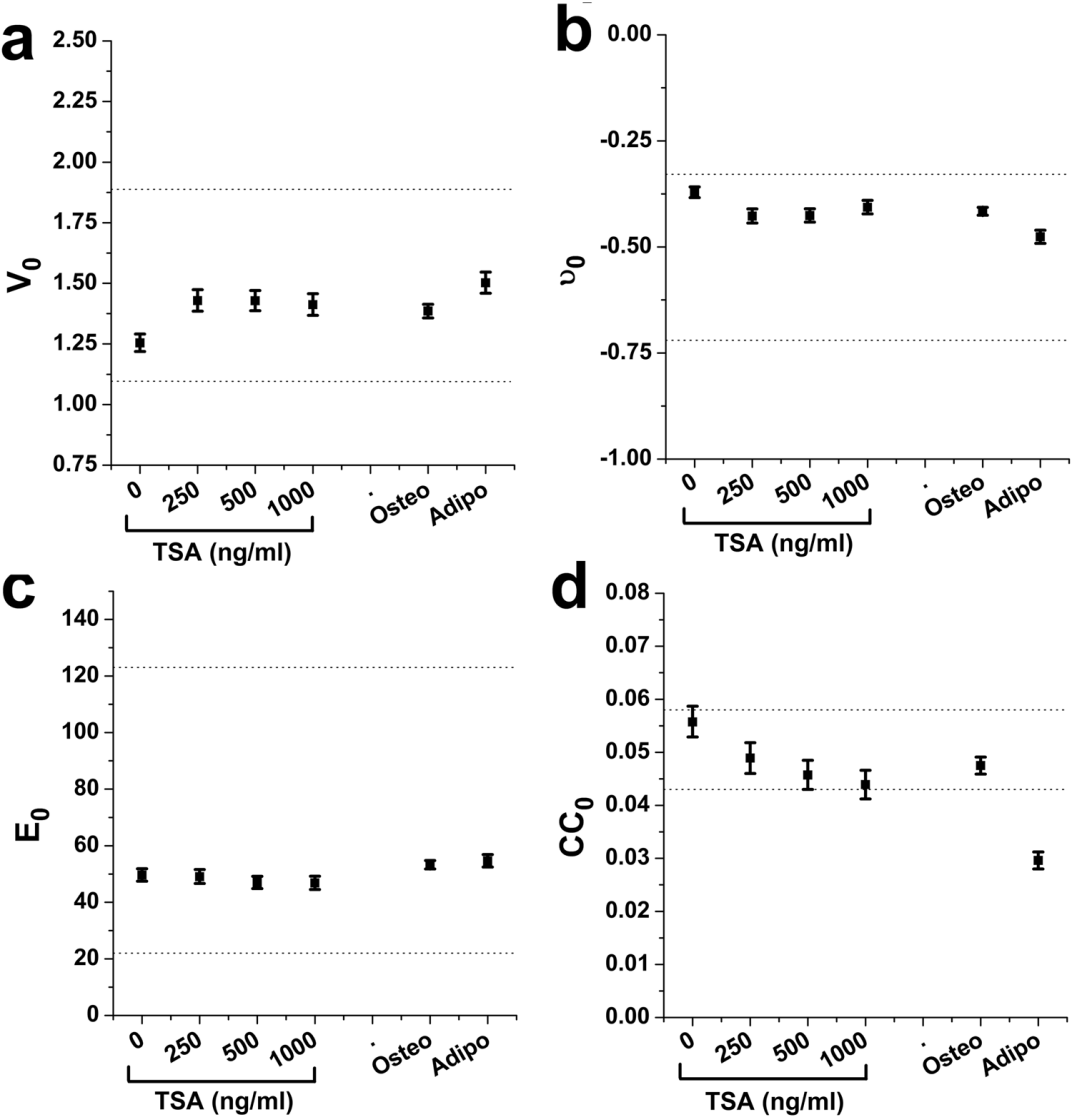
Chromatin decondensation and cellular differentiation affect nuclear state. Plot shows population average values for nuclear volume (**a**), Poisson’s ratio (**b**), apparent elastic modulus (**c**) and chromatin condensation (**d**) for increasing dosages of TSA or two well-established hMSC differentiation treatments (adipogenic and osteogenic differentiation). Dotted lines correspond to maximum and minimum values reachable via CSK-based modulation of nuclear state as obtained in Fig. 2, they are included to aid comparison.

We wanted to compare the established influence that the cytoskeleton plays on nuclear state versus internal influences, such as those associated with chromatin organization. To do so, we used TSA, a well-established drug to induce chromatin decondensation. Previous studies have shown that, in addition to causing chromatin to decondense, TSA also increases nuclear volume^28^, decreases Poisson’s ratio^9^ and reduces nuclear stiffness^8, 29, 30^. Importantly, it has been pointed out that long-term exposure to TSA (24 hours) may cause large cellular morphological changes, with cells displaying larger cell areas and multiple long extensions, as compared to untreated cells^31^. Indeed, in our hands TSA treatment lead to marked increases in cell spread area (not shown). Therefore, we avoided the confounding effects of TSA on cellular morphology and chromatin organization by fitting data sets as e.g. *E* = ${E}_{0}{[\frac{CSA}{\langle \overline{CSA}\rangle }]}^{\alpha }$. We use an analysis of covariance approach in which we force α to be equal for all TSA dosages. By doing so, we can single out the effect of chemically disrupting chromatin organization (changes in parameter E_0_) against the nuclear modulation associated with the TSA-induced increase in cell spread area (changes in parameter α). Accordingly, we compute and report E_0_, ν_0_, CC_0_ and V_0_ for each TSA dosage. As expected, when we treat our cells with increasing doses of TSA we observe decreasing values of chromatin condensation and apparent elastic modulus, in addition to increases in nuclear volume (Fig. 6). Surprisingly, the correlation between nuclear volume and chromatin condensation that we confirmed before was lost in cells treated with TSA (β = 0.06 ± 0.04; *p* = 0.07). It should be noted that the effect of TSA treatment in the measured nuclear parameters was somewhat limited, reaching ~13% increase in nuclear volume and ~6% decrease in apparent elastic modulus when the highest dose of TSA was used. If we combine the results obtained in Table 1 and in section 2.3, we predict that a 3-fold increase or a 1.3-fold decrease in cell spread area would give rise to similar changes in nuclear volume or elastic modulus, respectively.

We also compared the regulatory effect of the cytoskeleton on nuclear state against long-term changes such as those induced by cellular differentiation. To do so, we treated our cells for 21 days with well-known soluble factors to induce adipogenic and osteogenic differentiation. In this case, the changes observed were larger than those attained with TSA and of the same magnitude to those attainable through cytoskeletal reorganization (Fig. 6). Taken together, these results highlight the mechanical influence that the cytoskeleton has on the nucleus, and stress that the mechanical milieu of the cell plays a role comparable to internuclear factors on the regulation of nuclear state and function.

### The observed modulation of nuclear mechanical state by cell spread area extends to other mammalian cell types

**Figure 7:**
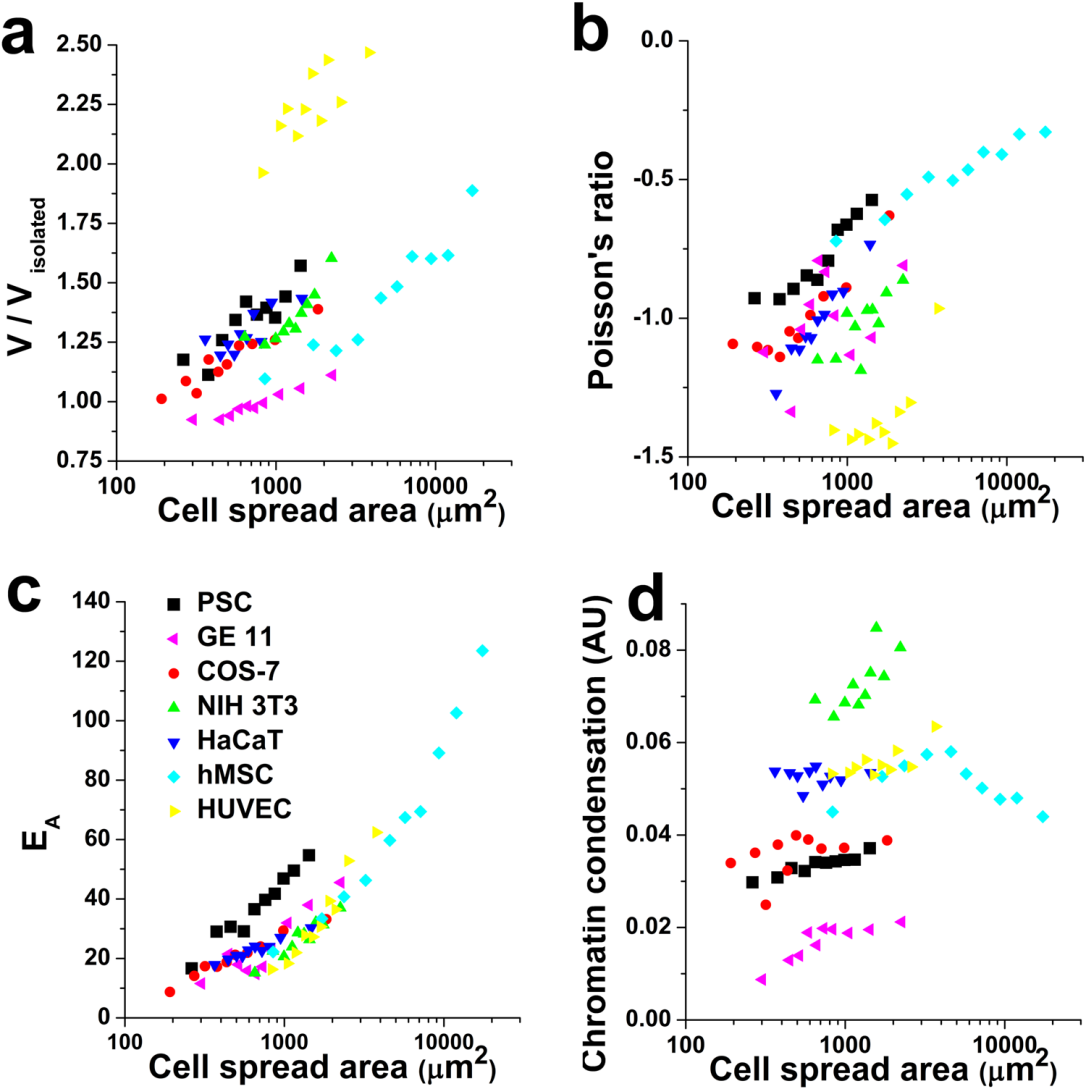
Cell spread area modulates nuclear state and mechanics in a variety of adherent cell types. Plot shows results for nuclear volume (**a**), Poisson’s ratio (**b**), apparent elastic modulus (**c**) and chromatin condensation (**d**) for the following cell types: hMSC (*light blue diamonds*), COS-7 (*red circles*), NIH 3T3 (green triangles), HaCaT (*dark blue triangles*), HUVEC (yellow triangles), PSC (*black squares*) and GE 11 (*pink triangles*). Values for >10 cells were pooled to compute each individual data point. Data is presented as geometric mean but error bars have been omitted for visual clarity. For the large majority of data points, the coefficient of variance computed as (Q1–Q3)/(2·Q2) was found to be smaller than 20%. For the single case of Poisson’s ratio, data is presented as mean.

Finally, we wanted to assess whether the mechanical interplay between the cytoskeleton and the nucleus could also be observed in other mammalian cell types. To do so, we extended our analysis to a variety of cell types, spanning different mammalian origins, tissue sources and degrees of proliferative capacity (primary and immortalized). Of note, in all cell types tested nuclei displayed auxetic behaviour, potentially extending to specialized cells the mechanical behaviour initially proposed by others as a potential hallmark of stemmness^9^. Furthermore, when comparing cell spread area versus nuclear mechanical properties, we recover similar trends to those initially observed in hMSC. In brief, larger cell areas tend to result in nuclei that are larger in volume (Fig. 7a), stiffer (Fig. 7c) and less auxetic (Fig. 7b). Conversely, the strong data overlap among different cell types is lost when plotting chromatin condensation vs cell spread area (Fig. 7d). Importantly, while the trends of the data are preserved, the different offsets in chromatin condensation highlight the fact that chromatin state is predominantly regulated by genetic and epigenetic mechanisms, while cellular spreading likely plays a second order modulatory effect.

It should be noted that the nucleoskeleton and the LINC complex are both dynamic mechano-responsive structures. For example, application of force on isolated nuclei via nesprin-1 triggers nuclear stiffening in the course of tens of seconds^32^. Said stiffening is mediated by the phosphorylation of emerin, a protein located in the inner nuclear membrane. Similarly, application of shearing forces onto isolated nuclei leads to unfolding of laminA’s immunoglobulin domains^3^, suggesting that tension applied onto laminA/C reduces its phosphorylation levels and promotes nuclear lamina assembly^33^. Given the short timescales required for mechano-responsive events to take place within the nucleoskeleton, it is likely that the changes we observe in cytoskeletal organization are accompanied by changes in nuclear lamina organization, which would then lead to the changes in nuclear mechanics. While it is beyond the aim of this study to assess whether changes in cytoskeletal state give rise to conformational changes in nucleoskeletal proteins, it is clear that the cytoskeleton can modulate nuclear mechanics. It now remains to be established whether this modulation takes place directly as a mechanically-dominant scaffolding structure surrounding the nucleus or indirectly through the regulation of mechanotransduction pathways that promote the assembly of proteins in the nuclear lamina.

### The nucleus may display distinct mechanical phenotypes according to cellular state and function

A body of research has focused on characterizing the mechanical properties of the cell’s nucleus, to understand its role on cellular fate and function. Our results contribute to the growing understanding that the nucleus is an auxetic material^9^, that displays strain-hardening^8, 14^ and visco-elastic^3, 34^ behaviours. The primary role of the nucleus is containing the cell’s genetic material, and as such it has to serve as a protective milieu in the event of external insults such as mechanical loads. Accordingly, it has been suggested that the larger nuclear stiffness observed for cells in stiffer tissues protects the nuclear material from the high stresses typically experienced in muscle, heart and bone^33^. Furthermore, the viscoelastic properties reported by others and the strain-hardening response suggested by our results may constitute additional mechanisms to reduce disruption of chromatin territories and DNA damage in stiff tissues. However, being the largest cellular organelle, the nucleus has to readily deform when the cell migrates. Therefore, the ability to decrease its volume when subjected to compressive loads may help the nucleus migrate through confined spaces. In addition, our results suggest that increased auxeticity is accompanied by decreased nuclear and cellular stiffness. These characteristics may thus define a migration-supportive mechanical phenotype that can be employed by nuclei. Taken together, these results suggest that the mechanical regulation facilitated by the cytoskeleton may allow the nucleus to dynamically engage in distinct mechanical phenotypes, as required by cellular function and the ever changing extracellular environment.

## Methods

### Cell culture

The majority of measurements were performed in human bone marrow derived mesenchymal stem cells (hMSCs), obtained from a commercial source (Stemcell Technologies, Cambridge, UK). Additional cell types were used for experiments presented in Fig. 7. In brief, COS-7, NIH 3T3, HaCaT and HUVEC cells were kind gifts from various researchers. Pancreatic stellate cells (PSCs) were derived from a C57/B6J mouse using a Histodenz gradient method derived from published protocols^35^. Immunostaining (DAPI + phalloidin) images of GE11 epithelial-like immortalized cells were provided courtesy of Dr. Guatrot’s group (QMUL). These cells had been stained and imaged using the same equipment and protocols described throughout the manuscript. The cell line had been initially generated by Sonnenberg’s lab using published protocols^36^. hMSC, COS-7, HaCaT and NIH 3T3 cells were cultured in media consisting of Dulbecco’s Modified Eagle Media (DMEM; Gibco, Paisley, UK) with 10% foetal bovine serum (FBS), penicillin (100 U/mL)-streptomycin (100 µg/mL; all Sigma-Aldrich, Dorset, UK). hMSCs were additionally supplemented with 1 ng/mL fibroblast growth factor-2 (FGF-2; PeproTech, London, UK). A subset of hMSCs cells were induced to differentiate for 21 days towards osteogenic or adipogenic lineages. For these cells, media was supplemented with Dexamethasone (100 nM), β-Glycerophosphate (10 mM) and L-ascorbic acid (50 µM) for osteogenic differentiation, or Dexamethasone (1 µM), IBMX (500 µM) Indomethacin (100 µM) and Insulin (10 µg/mL) for adipogenic differentiation. hMSCs between passages 3 and 8 were used for those experiments. HUVECs were cultured in M199 media supplemented with 10% FBS, 1 ng/mL EC growth factor-β, 3 µg/ml EC growth supplement from bovine neural extract, 1.25 μg/mL thymidine, 10 μg/mL heparin, 100 U/mL penicillin, and 100 mg/mL streptomycin. Cells were routinely passaged in tissue culture flasks, but were transferred to petri dishes containing glass coverslips prior to experiments. When transferred to coverslips, the media of HaCaTs was changed to Keratinocyte-SFM (ThermoFisher) supplemented with 50 ng/mL Bovine Pituitary Extract and 50 ng/mL Epidermal Growth Factor. This prevented the formation of cell clusters and enabled single cell imaging. Seeding densities between 900 and 5000 cells/cm^2^ were used in coverslips to guarantee that individual cells could be imaged. Seeding density was tailored to each cell type, and accordingly cells with smaller cell spread areas were seeded at higher densities. All pharmacological treatments and immunostaining procedures were carried out at least 2 days after cell seeding onto coverslips to guarantee optimal cell attachment and spreading.

### Pharmacological treatments and immunostaining

All reagents listed below were obtained from Sigma-Aldrich unless stated otherwise. A variety of chemical agents known to disrupt cytoskeletal filaments were used as follows: Blebbistatin, to inhibit Myosin II activity (5 µM, 20 µM and 50 µM), Withaferin A, to depolymerize vimentin filaments (0.5 µM, 1 µM and 2 µM) and Nocodazole to depolymerize microtubules (0.5 µg/ml, 1 µg/ml and 2 µg/ml). In addition, we used TSA to induce chromatin decondensation (250 nM, 500 nM and 1000 nM). Treatments were carried out for 1 hour for Blebbistatin, 3 hours for Withaferin-A, 2 hours for Nocodazole and 24 hours for TSA, directly on petri dishes containing cell-seeded glass coverslips. After the incubation period, cells were fixed and stored at 4 °C prior to immunostaining. For immunostaining experiments, cells were fixed with 3.7% Paraformaldehyde for 30 min and permeabilized with 0.1% Triton-X for 5 min. Cells were then treated overnight with primary antibodies against myosin (1:200 dilution Anti-myosin IIa rabbit monoclonal, M8064 Sigma), vimentin (1:400 dilution; Vimentin RV202 mouse monoclonal, Santa Cruz Biotechnologies), or α-tubulin (1:50 dilution; α Tubulin TU-02 mouse monoclonal, Santa Cruz Biotechnologies) diluted in goat serum blocking buffer at 4 °C. The next morning, coverslips were washed 3× with PBS and treated with FITC-tagged secondary antibodies (1:200 dilution, goat anti-mouse IgG-FITC, Santa Cruz Biotechnologies or goat anti-rabbit IgG-FITC, Sigma) and TRITC-tagged phalloidin (1:1000) for 1 hour at room temperature. Coverslips were washed again 3× with PBS and mounted on glass slides using ProLong® Gold Antifade Mountant containing DAPI (Thermo Fisher).

### Imaging

Immunostained cells were imaged using an inverted epifluorescence microscope (Leica DMI4000B) with a × 20/0.50 NA objective lens and a CCD camera (Leica DFC300FX). Only cells that appeared not damaged (based on the FITC and TRITC channels) and non-mitotic (based on the DAPI channel) were selected. The FITC channel was used for cell selection and coarse focusing. Cells were then sequentially imaged on the DAPI, FITC and TRITC channels, performing fine refocusing and adjusting the exposure time if necessary to guarantee optimal imaging conditions. In addition, a subset of cells was later imaged again using a laser scanning confocal microscope (Leica TCS SP2) with a × 40/1.25 NA oil immersion objective lens. For those cells, image stacks containing >80 images were acquired for the DAPI channel to reconstruct the 3D shape of nuclei.

### Quantification of cytoskeletal structures from fluorescence images

The algorithm for quantification of CSK structures at single cell level has been described in detail elsewhere^23^. Briefly, the algorithm uses grey-scale immunostaining-based images and it follows three independent steps: (1) initial fibre segmentation, (2) fibre refinement, and (3) determination and subtraction of background. The algorithm outputs a variety of parameters and maps that characterize the CSK structures of an individual cell. In particular, each pixel in the output map indicates either the brightness of segmented fibre or its local orientation. Information on maps is further simplified into individual descriptors such as cell spread area, total fibre amount, fibre thickness, global fibre alignment and fibre curvature. For the present study, we have used 3 parameters, namely: (1) ‘total fibre amount’, which quantifies the amount of protein building up the segmented cytoskeleton and is thus plotted and referred to as ‘CSK amount’ throughout the manuscript; (2) fibre anisotropy, which quantifies whether fibres are randomly oriented (value close to 0) or aligned in a parallel organization (value close to 1); (3) fibre main orientation, computed as the circular mean of the angular direction measured for all the fibres in the cytoskeleton. In our hands, the algorithm works equally well with fluorescence images of actin fibres, microtubules and intermediate filaments (vimentin), and also at different levels of magnification (20× to 63× oil). It is worth mentioning that the parameter ‘CSK amount’ is obtained through the fluorescence intensities of the acquired images. Accordingly, for each cytoskeletal protein quantified, the computed ‘CSK amount’ values will be dependent on the brightness of the fluorescence dye or secondary antibody used. To be able to compare polymerization levels of all proteins in a single graph (such as in Fig. 3), for each tested protein we normalize ‘CSK amount’ values using the median ‘CSK amount’ value computed for the whole cell population.

### Estimation of nuclear deformation and mechanical parameters

To estimate nuclear mechanical parameters, we assumed that an isolated undeformed nucleus will display a perfect spheroidal shape. The forces the nucleus is subjected to within the cell will then deform it in all 3 dimensions, to adopt the ellipsoidal shape measured through our epifluorescence images. We estimated that isolated undeformed nuclei will, on average, display a radius $\bar{r}$ = 12.9 ± 0.4 μm (see Fig. 2 and results section for explanation on how that value was obtained). It was also assumed that I_T_ would correlate with the volume displayed by each nucleus in isolated conditions, and we thus used I_T_ measured for each nucleus as readout of their undeformed diameter (*r*) as

2 $${r}_{i}=\langle \bar{r}\rangle {(\frac{{I}_{Ti}}{\overline{\langle {I}_{T}\rangle }})}^{1/3},$$

Similarly, we could also compute the undeformed volume (V_0_) for each individual nucleus, using:

3 $${V}_{0}=\frac{4}{3}\pi {r}^{3},$$

which could be compared to the actual nuclear volume measured as:

4 $$V=\frac{4}{3}\pi abc,$$

using the semi-axes values obtained as described in Supplementary Methods.

For each nucleus, Poisson’s ratio (*ν*) was obtained using the following relationship between volumetric change (ΔV) and length change (Δ*L*) for a stretched material:

5 $$\frac{{\rm{\Delta }}V}{V}={(1+\frac{{\rm{\Delta }}L}{L})}^{1-2\upsilon }-1,$$

Finally, to estimate a measure of the stiffness of the nuclei, we have slightly modified an approach presented by others^14^. Accordingly, we assume nuclei experience compressive loads in two main directions: normal compression which makes the nucleus flatten in the z axis, and lateral compression, which causes the nuclei of very elongated cells to display an elongated shape in the x-y axis. Based on their model, elastic modulus (*E*) is computed as:

6 $$E=\frac{{R}_{0}}{A}\frac{{F}_{C}}{{\rm{\Delta }}R},$$

Where *R*_*0*_ is the initial radius of the nucleus and *F*_*C*_ indicates the compressive intracellular forces applied by the cytoskeleton onto the nucleus. *A* is the contact area between the nucleus and the cytoskeleton, and it is computed as the area of an ellipsoid with major semi-axes *a*, *b* and *c*. Finally, we assume that intracellular tension scales linearly with cell spread area^14^, which allows us to estimate *F*_*C*_ for each probed cell by measuring their cell spread area (CSA). To acknowledge the fact that we are not using direct estimates for intracellular force, we use the term apparent elastic modulus (E_A_), which is then measured as:

7 $${E}_{A}=\frac{{R}_{0}}{A}\frac{CSA}{{\rm{\Delta }}R}.$$

### Estimation of chromatin condensation

In our epifluorescence images, chromatin condensation can be observed as bright ~1.5 μm diameter speckles, which are especially evident in the nuclear regions thicker than the depth of focus of the objective lens^37, 38^ (Fig. 1D). To highlight them with respect to other higher-order changes in fluorescence intensity of the nucleus, we performed band-pass filtering on the images, selecting only features with fluorescence intensity changes in the (0.75 µm–2.5 μm) spatial range. It is worth mentioning that chromatin condensation is made evident by the presence of both speckles brighter than its local surrounding, as well as similarly-size dimmer spots (Fig. 1E). Therefore, as a measure of chromatin condensation (*CC*), we added up the absolute value of the band-passed pixel intensities, for all pixels corresponding to the area where the nucleus was thicker than the depth of focus. Finally, to account for difference in brightness between nuclei, the computed value for chromatin condensation was divided by the fluorescence intensity of the nucleus. Additionally, we measured also the amount of chromatin condensation only in the periphery of the nucleus by using a mask ring corresponding to the outermost 25% of nuclear projected area.

## Electronic supplementary material

Supplementary information
