# Supplementary material for: Actomyosin and vimentin cytoskeletal networks regulate nuclear shape, mechanics and chromatin organization
Source: Sci Rep. 2017 Jul 12;7:5219. doi: 10.1038/s41598-017-05467-x (PMC5507932; doi:10.1038/s41598-017-05467-x)
Supplement: Supplementary file 1 — Supplementary information [file 41598_2017_5467_MOESM1_ESM.pdf]

Supplementary material

Actomyosin and vimentin cytoskeletal networks regulate nuclear shape, mechanics and chromatin organization

Michael C. Keeling[1], Luis R. Flores[1], Asad H. Dodhy[1] , Elizabeth R. Murray[2] & Núria Gavara[1*]

[1]School of Engineering and Materials Science, Queen Mary University of London, Mile End Road, E1 3NS, London, UK.

[2]Kinase Biology Laboratory, John Vane Science Centre, Barts Cancer Institute, Queen Mary University of London, Charterhouse Square, London EC1M 6BQ, UK.

[*]e-mail: n.gavara@qmul.ac.uk

Supplementary Method. Low magnification fluorescence imaging as a simple approach to estimate the coarse 3D shape of nuclei in adherent cells

The nuclei of adherent cells tend to display an ellipsoidal shape, with the direction perpendicular to the cell's substrate usually corresponding to the smallest semi-axis of the ellipsoid (which will be referred to as *c* semi-axis). Routine immunostaining images obtained in epifluorescence microscopes usually present a 2D projection of the nucleus, which is then used to quantify the dimensions of the two other semi-axes, parallel to the cell's substrate (which will be referred to as *a* and *b* semi-axis). Taking into account that dyes such as DAPI stain the whole nuclear interior, 2D projections still contain useful information of the z dimension. In detail, being a 2d projection, changes in the height of the nucleus as we move along the radial direction will give rise to changes in the fluorescence intensity measured. As a result, the intensity profile I(r) will have an elliptical-arc shape (Fig. 1, red line), mimicking the elliptical shape of the nuclear height profile. Unfortunately, this strategy is impaired by the reduced depth of focus of commonly used objective lenses (< 3µm) in comparison to the height of nuclei in adherent cells (> 3µm). Accordingly, when the imaged nucleus is considered to be in focus (usually when the largest projected area is obtained), the elliptical behaviour on the intensity profile I(r) is only recovered in the thinner regions, near the edge of the nucleus, while a rather flat intensity profile is obtained in the regions near the centre of the nucleus (Fig. 1A, black symbols). Of note, a similar phenomenon will be observed in any other dye-laden spheroidal structure as long as its height is larger than the depth of focus of the imaging objective. Accordingly, as an additional example, we show how a 4 µm-diameter fluorescent sphere (a Tetraspeck sphere typically used to calibrate confocal systems) will appear when imaged with a 60x oil objective (Supplementary Fig. 1). To measure the height of nuclei, we have taken advantage of the fact that, when using mid-range magnification objectives (20x), the number of pixels that still display a correct I(r) is sufficient to provide a good fit to an elliptical arc, thus allowing us to extrapolate the fluorescence intensity profile of the nucleus in thicker areas (Fig. 1A, red line). In addition, by integrating the ellipse recovered from the fit, we can also compute the total fluorescence intensity (I_T) that the nucleus would have displayed if all its depth could have been simultaneously imaged with the objective lens. Importantly, the estimated I(r) profile will relay the gross changes in nuclear height along the radial direction. Nevertheless, to carry out the next step and obtain the z(r) height profile of the nucleus, a conversion factor is needed to translate measured changes in fluorescence intensity into nuclear thickness. To obtain this conversion factor, we imaged a subset of cells using our standard approach and then obtained image stacks of the corresponding nuclei using a confocal microscope. In particular, we have measured and compared the following ratios between the ellipsoid semi-axes, using images of the same nuclei from epifluorescence and confocal microscopy:

$$\frac{c_{conf}}{\sqrt{a_{conf} \cdot b_{conf}}} \ vs \ \frac{I_0}{\sqrt{a_{epi} \cdot b_{epi}}} \quad , \quad (1).$$

where I_0 corresponds to the fluorescence intensity estimated from the fits (Fig. 1A red line) at the highest point of the ellipse, and the *conf* and *epi* subindices indicate the dimensions of semi-axes as estimated from the confocal or epifluorescence images, respectively. In parallel, to measure *a* and *b* semi-axes, we used the standard approach of thresholding the image of the nucleus to identify the nuclear outline. The outline was then fitted by an ellipse, whose major and minor semi axes corresponded to *a* and *b.* As shown in Supplementary Fig. S2, the calibration plot displayed a linear behavior with a good fit (r^2 = 0.98), indicating the reliability of our method. The slope of the fit corresponded to the conversion factor sought, which then allowed us to estimate *c* axis for each individual nucleus once I_0, *a* and *b* had been measured using exclusively the epifluorescence images. We also obtained similar fits comparing *a* and *b* axis values obtained using epifluorescence vs confocal images, thus verifying our ability to faithfully recover all nuclear dimensions. This is further verified by the high accuracy with which we can estimate nuclear volume. In particular, when computed as $\frac{V_{conf}-V_{epi}}{V_{conf}}$, we obtain an average value of 0.006 ± 0.1 (mean ± SD), indicating that most of the nuclei imaged had <10% discrepancy (over or underestimation) between the volume estimates obtained from confocal and epifluorescence images.

Given that our measurements are routinely performed in glass coverslips that have been mounted to glass slides using a hard-setting mountant, we verified that the mounting and curing procedure did not alter the shape of the studied nuclei. To do so, we stained fixed cells with DAPI (10 µg/ml in PBS for four minutes) and after thorough washing we imaged their nuclei using the same conditions described before. Subsequently, we mounted the cells with the same mountant used in our experiments (but without DAPI) and allowed 48 hours for mountant curing. Finally, the same nuclei population was imaged again using the same imaging conditions. After data analysis, we found no significant differences for nuclei dimensions when comparing population averages before (N = 121) and after (N = 171) adding mounting media. In particular, average nuclear axis data were *a:* 11.54 ± 1.56 µm vs 11.51 ± 1.7 µm (p = 0.96), *b:* 7.64 ± 1.4 µm vs 7.38 ± 1.5 µm (p = 0.18) and *c:* 4.54 ± 1.00 µm vs 4.55 ± 0.66 µm (p = 0.64).

As further verification, we analysed a large number of 4 µm-diameter Tetraspeck calibration spheres using the same approach and find an accurate measurement of their diameter in x, y, and z dimensions (Supplementary Fig. 1). It should be noted that the conversion factor in Supplementary Fig. S2 is only valid for our combination of imaging system and fluorescent dye. Accordingly, a new

conversion factor should be obtained using the same procedure if changes were made to any of said imaging conditions.

Limitations of the approach and comparison to previous methods

Simultaneous measurements of nuclear shape and chromatin condensation can pose an imaging conundrum. Nuclear 3-dimensional shape is readily assessed using very low-magnification objectives with large depth of focus. Conversely most image quantification approaches to readily estimate chromatin condensation rely on confocal-based high-resolution images of a thin section of the probed nucleus[9,37]. The logical step towards simultaneous estimation of nuclear shape involves the acquisition of dense image stacks, a process that tends to be time-consuming, prior to volumetric reconstruction via commercial software. Notably, the process becomes even lengthier if simultaneous quantification of the cytoskeleton is also desired, thus limiting the number of cells/nuclei that can be tested. We have identified an advantageous middle ground in the use of mid-resolution imaging. Said approach requires only 1 image per cell, and allows us to visualize the central parts of the nucleus as sectioned (to estimate chromatin condensation) while in the nuclear periphery we visualize the graded intensity profile of a structure that is changing in thickness. While the approach is ripe for high-throughput imaging in combination with cytoskeletal quantification, it has some limitations. Firstly, we estimate the gross morphology of the nucleus, and we model it as an ellipsoid characterized by 3 semi-axes. Accordingly, we can’t make hypotheses on whether the large changes in volume that we observe are associated with wrinkling or unfolding of the nuclear envelope[38]. Secondly, our approach to estimate chromatin condensation is based on the presence of bright chromatin-rich structures in the µm-length scale. At the moment, we can’t account for higher-order chromatin structures, such as those resulting from buckling of the nuclear membrane, it is likely that this limitation is impacting our results on chromatin condensation for cells with very reduced spread areas (Fig. 2D).

Supplementary Note. Global fit to untangle the contribution of each cytoskeletal network

To perform the global fit, we use the fact that we have quantified actin amount for all probed cells, while the second cytoskeletal protein quantified was myosin, vimentin or tubulin. We group cells according to CSK protein stained (myosin, vimentin or tubulin) and for each nuclear parameter we compute 3 multivariate fits with two independent variables ([A] and [M]; [A] and [T]; or [A] and [V]), which take a form such as:

$$E = E_0 \left[\frac{A}{\langle \bar{A} \rangle}\right]^{\alpha_A} \left[\frac{M}{\langle \bar{M} \rangle}\right]^{\alpha_M}, \quad (3).$$

Where, for this partial fit example, only cells stained against actin and myosin are used. Finally, to achieve the global fit solution, we iteratively impose that the fitting constants obtained for actin amount and offset (α_A and E_0 respectively in the equations below) should be equal in all 3 multivariate fits. It should be noted that the values for cytoskeletal amount are obtained via fluorescence image quantification, and they are dependent on the concentration of primary and secondary antibodies used, as well as the brightness of the secondary antibody tag and the filter sets used in the microscope. This limits our ability to truly measure the relative amount of one cytoskeletal protein versus another. For that reason, prior to fitting we normalize all fluorescence quantification values using the predicted fluorescence intensities for cells with 5200 μm^2 spread area (computed median population value for cell spread area), according to Fig. 2. Using this approach, we can readily compare the values obtained for the fitting constants α_A, α_M, α_T and α_V as a faithful indication of the relative influence of each cytoskeletal network on nuclear mechanics. Finally, it should be noted that a cell displaying cytoskeletal amounts close to the population average will have values for e.g. $\left[\frac{A}{\langle \bar{A} \rangle}\right]$ close to 1. Therefore, the offset values found in our fits (E_0 for example) correspond to the value for nuclear properties of a cell representing the population average, rather than the limit with no cytoskeletal assembly.

Supplementary Figure S1

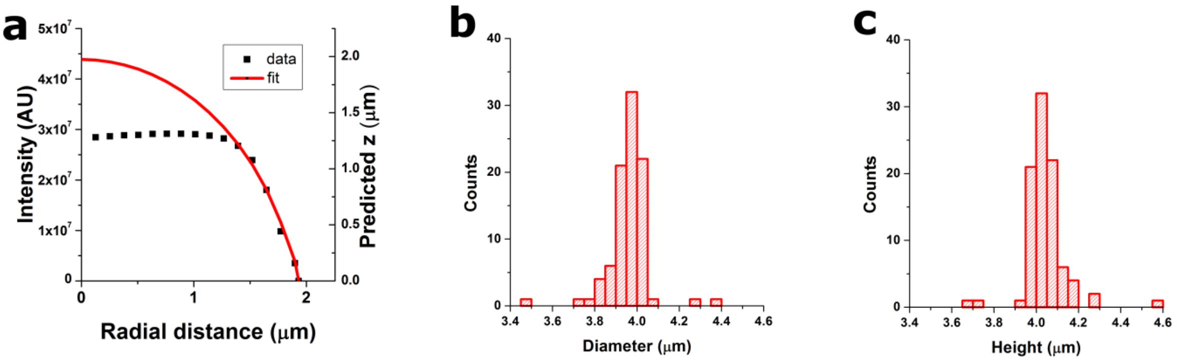

Verification of the method using fluorescent spheres of known constant diameter. 4 µm-diameter Tetraspeck beads were imaged using a 63x oil objective, to reproduce the conditions in which the depth of focus of the imaging objective is smaller than the height of the imaged object. (*a*) Averaged fluorescence intensity profile as a function of radial distance I(r) for one example sphere. Black squares correspond to fluorescence intensities recorded. Red line corresponds to the ellipse obtained when fitting the fluorescence intensity profile of the outermost pixels. More than 100 Tetraspeck beads were imaged and analysed. The histograms in (*b*) and (*c*) present the distribution of estimated diameters and heights, respectively.

Supplementary Figure S2

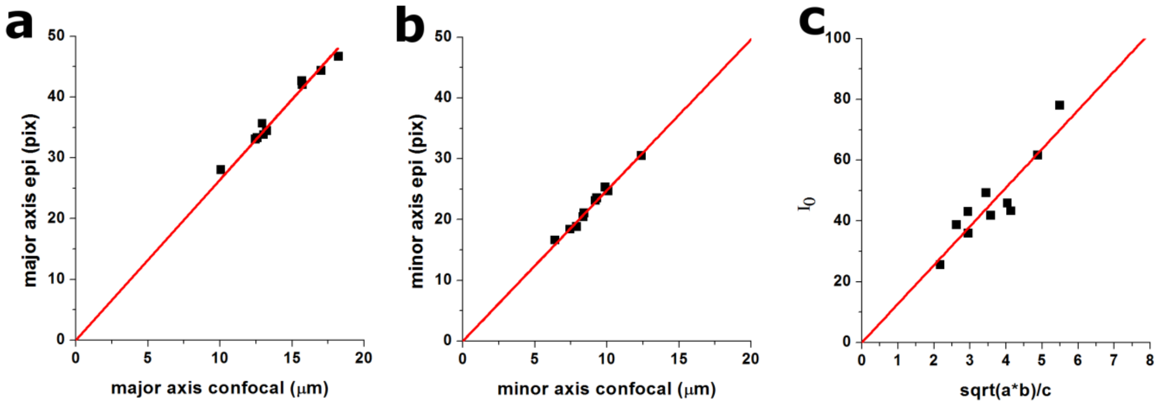

Comparison between ellipsoid dimensions obtained from confocal and epifluorescence imaging. Plot shows results for (*a*) major axis (r^2 = 0.999), (*b*) minor axis (r^2 = 0.999), and (*c*) z axis (r^2 = 0.98). Each data point corresponds to an imaged nucleus.

Supplementary Figure S3

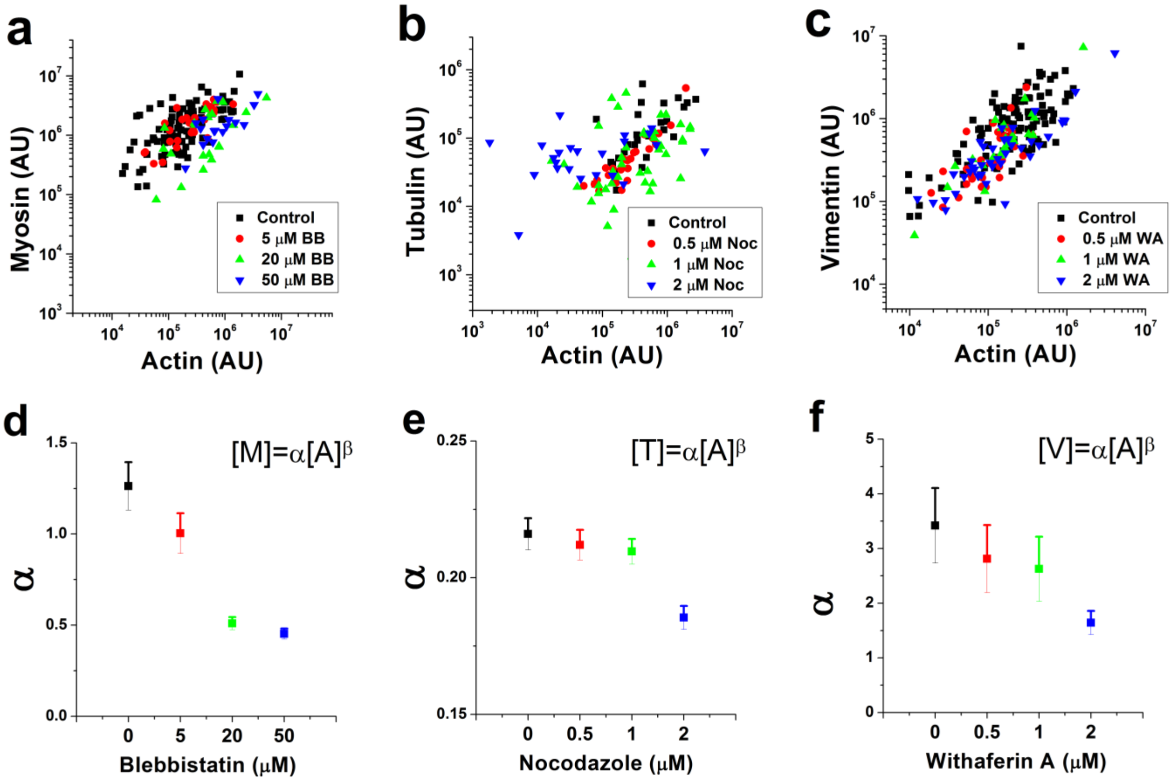

Values for CSK assembly are correlated among different cytoskeletons. Upper row shows results for actin vs. myosin (*a*), actin vs. tubulin (*b*), and actin vs. vimentin (*c*). Symbols with different colours indicate different dosages of pharmacological treatments to disrupt a cytoskeleton. Each data point corresponds to a cell. For each dosage, data was fitted using a power fit as shown in the lower row, to obtain the slopes (α) that show the effect of increasing dosages of Blebbistatin (*d*), Nocodazole (*e*) and Withaferin A (*f*).
